# Human alveolar echinococcosis in Slovakia: Epidemiology and genetic diversity of *Echinococcus multilocularis*, 2000–2023

**DOI:** 10.1371/journal.pntd.0011876

**Published:** 2024-01-10

**Authors:** Daniela Antolová, Viliam Šnábel, Júlia Jarošová, Serena Cavallero, Stefano D’Amelio, Yaroslav Syrota, Róbert Rosoľanka, Mária Avdičová, Martina Miterpáková

**Affiliations:** 1 Institute of Parasitology of the Slovak Academy of Sciences, Košice, Slovakia; 2 Department of Public Health and Infectious Diseases, Sapienza University of Rome, Rome, Italy; 3 I. I. Schmalhausen Institute of Zoology of National Academy of Sciences of Ukraine, Ukraine, Kyiv, Ukraine; 4 Clinic of Infectology and Travel Medicine, Jessenius Faculty of Medicine and University Hospital in Martin, Comenius University Bratislava, Martin, Slovakia; 5 Regional Authority of Public Health Banská Bystrica, Banská Bystrica, Slovakia; Istituto Superiore Di Sanita, ITALY

## Abstract

Human alveolar echinococcosis (AE) is a serious parasitic disease caused by larval stages of *Echinococcus multilocularis*. Between January 2000 and October 2023, 137 AE cases were confirmed in Slovakia. The average annual incidence increased from 0.031 per 100,000 inhabitants between 2000 and 2011, to an average of 0.187 since 2012, i.e. about six times. Among patients, 45.3% were men and 54.7% were women; the mean age at the time of diagnosis was 52.8 years. Most cases were diagnosed in the age groups 51–60 years and 61–70 years (33 cases each), and eight patients fell into the age category ≤ 20 years. To better recognize the gene diversity in clinical samples, metacestodes from 21 patients collected between 2013 and 2021 were subjected to DNA sequencing of four mitochondrial genes. Using concatenated sequences of *cob* (603 bp), *nad2* (882 bp) and *cox1* (789 bp) gene fragments, 14 isolates (66.7%) were assigned to the European E5 profile of *E*. *multilocularis*, two isolates (9.5%) to the E5^a^ subtype, four isolates (19%) to the E4 profile, and one isolate (4.8%) to haplogroup E1/E2. The E5-type profiles and E4 profiles were distributed throughout the country, whereas the E1/E2 profile was found in the patient from western Slovakia. According to the data obtained and GenBank sequences, the E5-type dispersal is so far limited to central-eastern Europe and the variant seems to be indigenous to that region. The admixture with the haplotypes E4 and E1/E2 could have taken place from a historical endemic focus during the fox expansion in the last decades. By employing the *nad1* fragment, a typical European haplotype was observed in all 21 resolved Slovak samples. The acceleration in the AE incidence in the last decade suggests the emergence of the disease and the need for further research on human and animal isolates.

## Author summary

Human alveolar echinococcosis (AE) is a serious parasitic disease that may lead to death within 10–15 years after diagnosis if untreated. It is caused by larval stages of the fox tapeworm, *Echinococcus multilocularis*, which predominantly circulates in the sylvatic cycle, with carnivores (foxes, raccoon dogs, jackals, wolves) as definitive hosts and small mammals as intermediate hosts. Human infection occurs after the accidental ingestion of infective parasite eggs. The liver is the most often affected organ, and the parasite shows features similar to malignant tumors with invasive and infiltrative growth and metastasis formation. Here we present current epidemiological data on human alveolar echinococcosis in Slovakia and compare the geographical distribution of AE cases with occurrence of parasite in red foxes. Moreover, the genetic diversity of *E*. *multilocularis* is not yet fully understood, and it is supposed that even subtle genetic differences may have relevance for variation in infectivity for humans. Therefore, we addressed the question of distribution of *E*. *multilocularis* haplotypes in clinical isolates across the country and investigated their genetic relatedness with haplotypes in other countries of Europe.

## Introduction

Human alveolar echinococcosis (AE) is a serious zoonotic disease with a considerable socioeconomic impact. The disease caused by larval stages of *Echinococcus multiloculari*s can exceed fatality rate of 90% within 10–15 years after diagnosis if left untreated [1]. The primary infection develops almost exclusively in the liver and manifests as slowly progressing disease with a tumour-like, multivesicular infiltrative structure, responsible for tissue destruction [2,3]. At a later stage, metastases can be formed *per continuitatem* in organs adjacent to the liver (gallbladder, pancreas, diaphragm, etc.), or by haematogenous or lymphatic route in more distant structures (lungs, bones, brain, etc.) [4].

*E*. *multilocularis* is predominantly transmitted in a sylvatic cycle with carnivores (foxes, raccoon dogs, jackals, wolves) as definitive hosts and small mammals as intermediate hosts. Domestic carnivores, predominantly dogs, can also be infected and their contribution to environmental contamination with *E*. *multilocularis* eggs in central Europe typically ranges between 4% and 19% [5]. Humans are considered dead-end hosts and acquire the infection by accidental ingestion of eggs released by infected definitive hosts to the environment [4]. AE is currently recognized as one of the most dangerous parasitic zoonoses in large parts of the Eurasian continent [6]. In recent decades, human AE has emerged in central and eastern Europe, which is probably associated with the growing population of foxes and their progressive invasion of urban and peri-urban environments [7].

Slovakia, located in this part of Europe, is currently considered a country with an endemic occurrence of *E*. *multilocularis*. An extensive full-scale survey on more than 4,700 red foxes conducted between 2000 and 2010 showed a 30.3% average prevalence rate of the parasite and the existence of highly endemic localities in some northern areas of the country with 50–60% positive animals [8]. Human AE cases has begun to regularly occur in Slovakia at the beginning of this century and since then the number of patients is steadily increasing [9,10].

The genetic diversity of *E*. *multilocularis* is not yet as clear-cut as in the closely related *Echinococcus granulosus sensu lato*, although subtle genetic differences may have relevance for variation in infectivity for humans [11]. Given its sylvatic origin and host species, a genetic subdivision of *E*. *multilocularis* was recorded in isolates obtained from North America, Europe, Mongolia and Asia, formerly by DNA sequencing of mitochondrial targets [12,13]. Questions still remain regarding the ambiguous epidemiological aspects of the parasite, with a hyper-endemic focus in China and only a few autochthonous clinical cases in North America, the dynamics of parasite transmission at local levels, and the distribution patterns of human mtDNA haplotypes circulating in Slovakia in association with the European context.

For Slovakia, only a few studies focused exclusively on isolates from animals (red foxes, grey wolves, dogs) were conducted on the genetic diversity of *E*. *multilocularis* [e.g., 14–17] Therefore, with the aim to contribute to an overview of the epidemiological situation by focusing on transmission to humans, the present study was conducted to evaluate the epidemiology of human AE and the haplotype diversity of *E*. *multilocularis* in clinical isolates from different parts of the country. For this purpose, four mitochondrial genes (*nad1*, *nad2*, *cox1*, *cob*) were examined and the sequence data were phylogenetically evaluated also with regard to a broader European picture, influenced by the recent parasite spread and emergence.

## Material and methods

### Ethics statement

The study was conducted in accordance with the ethical standards as laid down in the Declaration of Helsinki of 1975, as revised 2013, and approved by the Ethics Committee of the Institute of Parasitology of the Slovak Academy of Sciences (Protocol Code EK/05/2013, November 30, 2013 and EK/01/2018, December 14, 2018).

### Collection of biological material and evaluation of clinical data

In Slovakia, based on the Directive No. 626/2004 of the Slovak Republic (SR) Government on Monitoring of Zoonoses and Their Causative Agents, all confirmed cases of echinococcosis are reported to Public Health Authority of the Slovak Republic (PHA SR) operated by the Ministry of Health. In order to obtain the most accurate information, data on patients with AE were collected in cooperation with PHA SR and compared with data from clinics of infectious diseases, surgery, neurology, etc. from various areas of the country. In total, data on 137 human AE cases diagnosed in Slovakia between January 2000 and October 2023 were obtained. Only “confirmed” cases, as categorized by Brunetti and colleagues [2], were included into the study.

*E*. *multilocularis* isolates (abscess contents, metacestode tissues) for genetic analyses were collected during the diagnostic procedures or surgery interventions at specialized outpatient clinics throughout Slovakia. A total of 21 samples, specifically liver abscess contents (*n* = 12), metacestode tissues from liver (*n* = 6), brain lesion content (*n* = 1), lung abscess content (*n* = 1), and content of the abscess located in the lumbar region (*n* = 1) were collected between 2013 and 2021. The group of patients consisted of 11 men and 10 women (Table 1). In most cases (*n* = 18), native samples (liquid abscess contents) or samples stored in physiological solution were sent directly to the Institute of Parasitology, Slovak Academy of Sciences in Košice. In three cases, paraffin-embedded tissues were used.

**Table 1:** Characteristics of examined human isolates of *Echinococcus multilocularis*.

Isolate	Metacestode localization	Age of patient	Gender	Year of sampling	Geographical origin (district, region, location)
**SK1**	Liver	54	M	2020	Púchov (TN), NW
**SK2**	Brain	75	M	2020	Piešťany (TT), W
**SK3**	Liver	37	F	2019	Detva (BB), C
**SK4**	Liver	54	F	2018	Bardejov (PO), NE
**SK5**	Liver	18	F	2016	Sabinov (PO), E
**SK6**	Liver	51	F	2018	Martin (ZA), N
**SK7**	Liver	23	M	2017	Veľký Krtíš (BB), SC
**SK8**	Liver	60	M	2017	Kysucké Nové Mesto (ZA), NW
**SK9**	Liver	77	M	2013	Banská Bystrica (BB), C
**SK10**	Lumbar region	51	F	2016	Žarnovica (BB), C
**SK11**	Liver	44	M	2016	Levice (NT), S
**SK12**	Liver	15	M	2016	Tvrdošín (ZA), N
**SK13**	Liver	17	F	2015	Tvrdošín (ZA), N
**SK14**	Liver	75	F	2015	Svidník (PO), NE
**SK15**	Liver	51	M	2014	Stará Ľubovňa (PO), N
**SK16**	Lung	61	M	2013	Tvrdošín (ZA), N
**SK17**	Liver	26	F	2010	Poprad (PO), N
**SK18**	Liver	19	M	2012	Žilina (ZA), N
**SK19**	Liver	80	F	2015	Čadca (ZA), NW
**SK20**	Liver	51	F	2013	Považská Bystrica (TN), NW
**SK21**	Liver	52	M	2021	Senica (TT), W

M–male; F–female; TN–Trenčín region, TT–Trnava region, BB–Banská Bystrica region, PO–Prešov region, ZA– Žilina region, NT–Nitra region; N–north of Slovakia; S–south of Slovakia; W–west of Slovakia; E–east of Slovakia; C–central Slovakia.

To test the association between the incidence of echinococcosis in humans and the prevalence of this helminth infection in fox populations, a statistical analysis was performed in the R programming environment [18], by using the ´tidyverse´ collection of packages [19] for data manipulation and visualization. To ensure consistent comparisons between districts of Slovakia, incidence rates were standardized. This procedure involved dividing the total number of confirmed cases by the district’s population and multiplying the result by 100,000. The distribution of standardized rates of human cases was evaluated using the check distribution function from the ´performance´ package [20].

A Tweedie generalized linear model (GLM) was then applied that used the standardized number of human cases per 100,000 as the response variable and the prevalence of infection in foxes as the explanatory variable. The model also included weights represented by the number of foxes examined in each district to account for variations in fox sample sizes between the districts. For the implementation of the model, the glmmTMB function from its respective package was used [21]. The fit of the GLM was assessed by the coefficient of determination (R^2^), calculated using the r2 function from the ’performance’ package, which denotes the proportion of the variance explained by the model. Then, the predictive capability of the model was assessed via a posterior predictive check, conducted using the posterior predictive check function from the ’performance’ package.

### DNA extraction and amplification

Total genomic DNA from abscess contents or metacestode tissues was extracted using the DNeasy tissue kit (Qiagen, Hilden, Germany), according to the manufacturer’s instructions. The complete mitochondrial gene of NADH dehydrogenase 2 gene (*nad2*, 882 bp) and fragments of mitochondrial genes cytochrome *b* (*cob*, 603 bp), cytochrome *c* oxidase 1 (*cox1*, 789 bp) and NADH dehydrogenase 1 (*nad1*, 395 bp) were examined. DNA was amplified using specific primer pairs under the conditions described in Table 2. Positive PCR products were purified using ExoSAP IT PCR Express Product Cleanup Reagent (Thermo Fisher Scientific, USA) and sequenced in both directions using the same primers as those used for PCR amplifications.

**Table 2:** Primer sequences and annealing temperatures used for amplification of mitochondrial DNA in four genes of *Echinococcus multilocularis*.

Gene fragment/ ct gene	Primer designation	Primer sequence (5´- 3´)	Annealing temperature	Reference
***cob* (603 bp)**	cob-F	TGCTGATTTGTTAAAGTTAGTGATC	54°C	[22]
	cob-R	CATAAATCAATGGAAACAACAACAAG		
nad2* **(882 bp)**	nad2-F	GCGTTGATTCATTGATACATTGT	55°C	[12]
	nad2-R	TAGTAAAGCTCAAACCGAGTTCT		
***cox1* (789 bp)**	F1	TTGAATTTGCCACGTTTGAATGC	55°C	[23]
	R3	TTGAATTTGCCACGTTTGAATGC		
***nad1* (395 bp)**	Cest1	TGCGTTATTGGCATATGGTAG	60°C	[24]
	Cest2	GTGCCACCCTCAGTTGGTACT		

*nad2 –complete (ct) gene analyzed

### Sequence analysis and genetic data processing

Nucleotide sequences were manually checked and edited using the Chromas 2.6.6 program (Technelysium Pty Ltd.; http://www.technelysium.com.au/chromas.html). The BLASTn algorithm [25] was used to confirm the identity of each isolate. Sequences were clustered by similarity and aligned using the Clustal Omega tool [26]. To increase the complexity of the phylogenetic analysis, we have decided to evaluate concatenated data of *cob*, *nad2*, *cox1*, as these genes were commonly employed concomitantly in several surveys after the study of Nakao and colleagues published in 2009 [12], who first established a global geographic pattern of genetic variation in *E*. *multilocularis* including five European haplotypes (E1 –E5). Unlike the Nakao´s scheme where the complete *cob*, *cox1*, *nad2* genes were examined, we have sequenced the partial genes of *cob* (a length of 603 bp, corresponding to the informative section of 237–840 bp of the gene involving the polymorphic sites for E1 –E5) and *cox1* (789 bp, with the informative section of 324–1113 bp), whereas the *nad2* gene (882 bp) was completely sequenced. A fragment of the *nad1* gene (395 bp) was phylogenetically evaluated separately given that several studies on *E*. *multilocularis* employed solely this locus, or in combination with other gene, such as 12S rRNA [e.g., 27,28].

Phylograms were constructed using the MEGA 11 software [29] by the maximum-likelihood (ML) method with 1,000 bootstrap pseudoreplicates. The best-fitting nucleotide substitution models according to a hierarchical likelihood ratio test were chosen Hasegawa-Kishono-Yano (HKY) + G models for the concatenated *cob*, *nad2*, *cox1* sequences (2,274 bp), and HKY model for the *nad1* sequences (395 bp). Statistical parsimony networks estimated using the Median Joining network approach were depicted for the haplotype association analysis in the concatenated (*cob*, *nad2*, *cox1*) and *nad1* datasets. The networks were constructed using the PopART software according to Bandelt et al. [30].

Population diversity indices such as haplotype diversity (Hd), nucleotide diversity (Nd), average number of pairwise nucleotide differences within population (K) and neutrality index Tajima´s [31] were calculated using the Arlequin v. 3.5 population genetics software [32] and the MEGA11 software. They were used to estimate the degree of population differentiation and assess the history of recent demographic events in three European populations, somewhat arbitrarily defined by country: the Slovak samples here analyzed and the samples from Poland and France already published by Karamon et al. in 2017 [33] and Nakao et al. in 2009 [12], for which more sequence data were available in GenBank (*n* = 74 and *n* = 10, respectively). The two newly endemic European countries (Poland, Slovakia) and country with historically endemic areas (France) were thus included for the comparison of the genetic subdivision. For France, the exact geographical origin of fox samples was not specified in the Nakao´s study, but it is assumed that the major part of sampling took place in the historical focus defined in eastern France, spanning from the northeastern border to the Southern Alps, with a limited focus in the Massif Central [34], and/or in the close endemic areas formed during the recent westward and northward expansions [35].

To measure the genetic diversity in populations, which considers the number of haplotypes as well as the relative abundance of each haplotype, the inverse Simpson’s Diversity Index (1-D) [36] was also calculated.

The pairwise genetic difference for the investigated populations was estimated using Wright’s F-statistics (Fst), which measures the proportion of within-population variation to total variation, and was calculated by the Arlequin v3.5. Its value ranges from 0 (random distribution of alleles across populations) to +1 (no common allele between each considered sample). As suggested by Wright et al. [37], Fst values < 0.05 were rated as insignificant differentiation, 0.05 to 0.15 as moderate differentiation, 0.15 to 0.25 as large differentiation, and Fst > 0.25 as very large differentiation.

## Results

### Epidemiology of human alveolar echinococcosis in Slovakia

**Fig 1:**
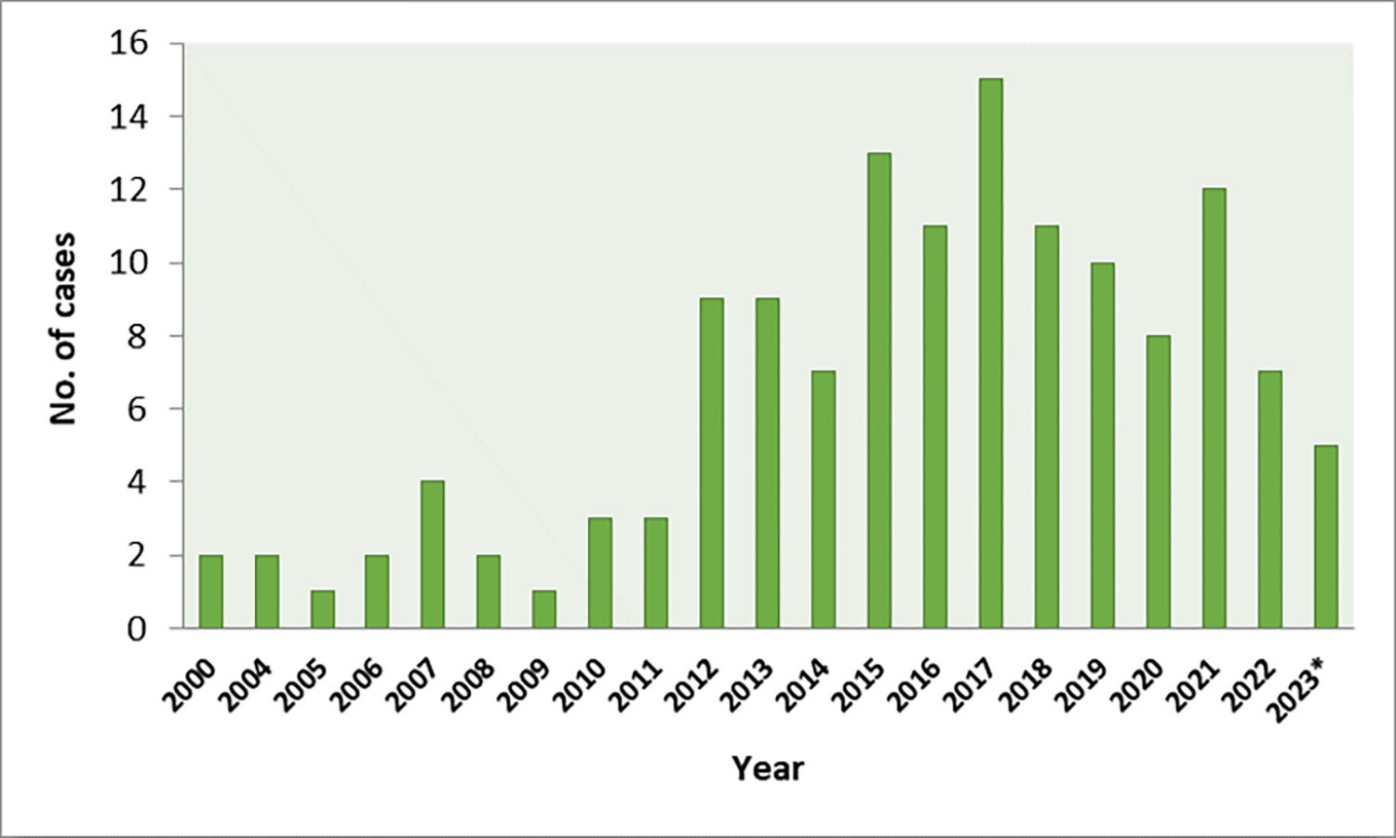
Human AE cases diagnosed in Slovakia between January 2000 and October 2023. *—cases reported by October 2023.

During the nearly 23-year-study period, 137 AE cases (listed in S1 Table) were diagnosed and classified as “confirmed” in Slovakia. One to four cases of the disease were reported per year between 2000 and 2011, which corresponds to the annual incidence of 0.031/100,000 inhabitants. Since 2012, the number of cases has increased to a level of between 7 and 15 per regular year, which corresponds to the annual incidence of 0.187/100,000 inhabitants (Fig 1).

Among patients, 62 (45.3%) were men and 75 (54.7%) women (M: F ratio = 0.83/1.0). Mean age at the time of diagnosis was 52.8 ± 17.4 years, ranging from 6 to 80 years. Specifically, in women the age varied from 6 to 80 years (mean 52.6) and in men from 8 to 80 years (mean 52.5). The disease was the most commonly diagnosed in the age groups of 51–60 years (33 cases) and 61–70 years (33 cases). Less frequently, AE was diagnosed in the age of 71–80 years (21 cases); 41–50 years (16 cases); 31–40 years (15 cases), and 21–30 years (11 cases). Eight (5.8%) patients were in the age category ≤ 20 years (specifically, 6, 8, 14, 14, 17, 18, 19, and 20-year-old patients).

Data on the localization of metacestode tissues were available in 89 patients. In 88 cases, the primary location was liver, while in an 8-year-old boy the right femur was primarily affected. In most patients– 77 (86.5%), the liver was the only affected organ, while in 12 cases metastases or infiltration to adjacent structures were reported. Secondary foci were detected in the lungs (4 cases), abdominal cavity (3 cases), and in the kidney, adrenal gland, spleen and adjacent lymph node, and in the brain in one case each.

**Fig 2:**
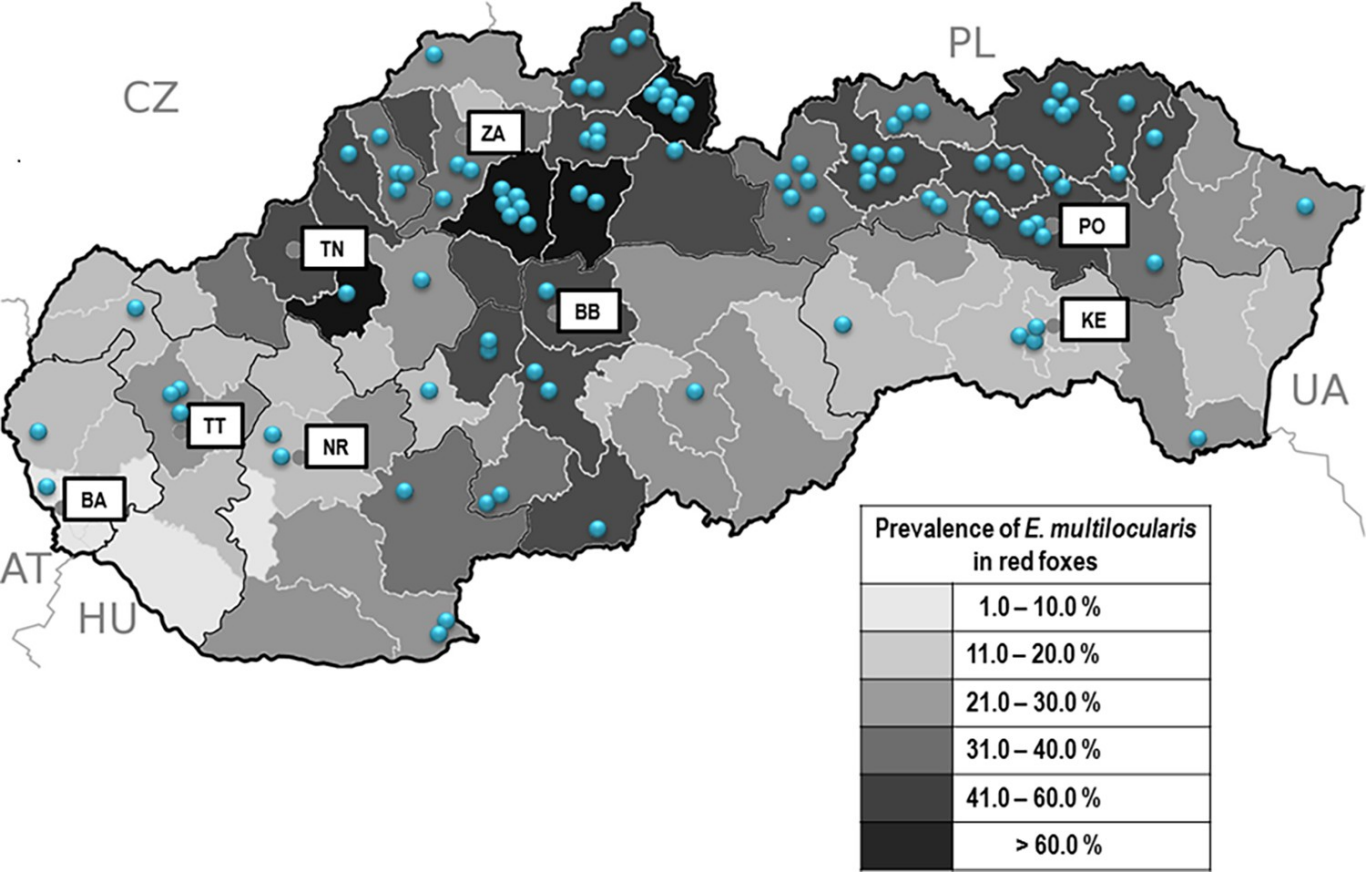
Geographic distribution of human cases of alveolar echinococcosis (blue dots) reported between January 2000 and October 2023, and prevalence of *Echinococcus multilocularis* in red foxes (grey scale) recorded in Slovak districts between 2000 and 2010. Abbreviations: AT–Austria, CZ–Czech Republic, PL–Poland, UA–Ukraine, HU–Hungary, BA–Bratislava region, BB–Banská Bystrica region, KE–Košice region, NR–Nitra region, PO–Prešov region, TN–Trenčín region, TT–Trnava region, ZA– Žilina region. The base map was used from public domain (https://commons.wikimedia.org/wiki/File:Slovakia_districts.png).

Information on the residences of patients was available in 95 cases. Geographically, their origin correlated with the prevalence of *E*. *multiloculari*s in red foxes as described by Miterpáková and Dubinský in 2011 [8]. In fact, most of the patients came from areas with a more frequent occurrence of parasite in foxes. Overall, 75 (78.9%) patients originated from districts where the prevalence of tapeworm in red foxes exceeded 30%, of which 15 patients were from districts with more than 60% of infected animals. On the other hand, only 20 (21.1%) patients came from districts where less than 30% of foxes were infected (Fig 2). The average incidence of AE in districts with more than 30% prevalence of *E*. *multilocularis* in red foxes was three times higher (0.09/100,000 inhabitants/year) than in districts with less than 30% infected foxes (0.03/100,000 inhabitants/year) (see also S2 Table).

**Fig 3:**
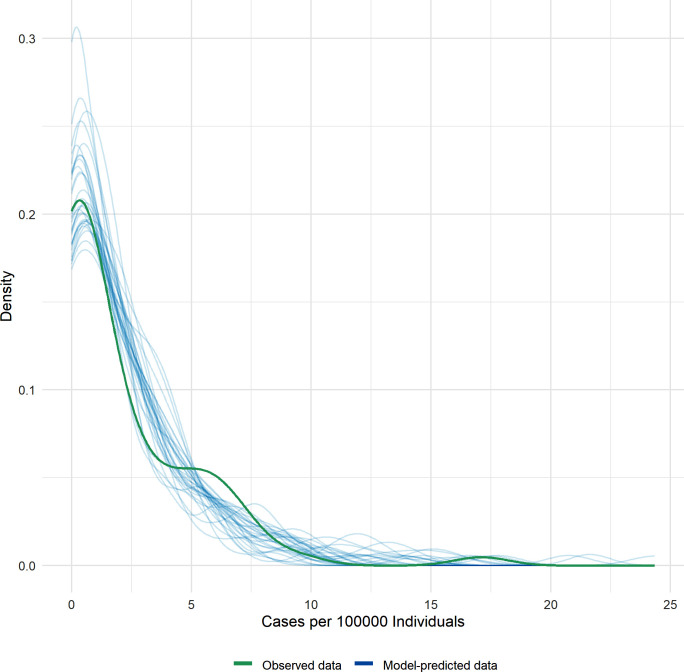
Posterior predictive check analysis. The green line represents the observed data, while the blue lines represent the model-predicted data (The model-predicted lines should resemble the observed data line).

The model approach also showed a significant positive association (β = 4.7, p << 0.001) between the occurrence of human echinococcosis and the prevalence of *E*. *multilocularis* in foxes at the district level. The model, having a marginal R2 of 0.58, suggested that approximately 58.3% of the variation in human cases is explained by the parasite prevalence. The posterior check (Fig 3) showed that the data align with the peak predictions of the model. Despite some discrepancies between the observed and predicted data, the adequacy of the model to represent the data satisfactorily was affirmed.

### DNA sequencing of cob, nad2, cox1

**Fig 4:**
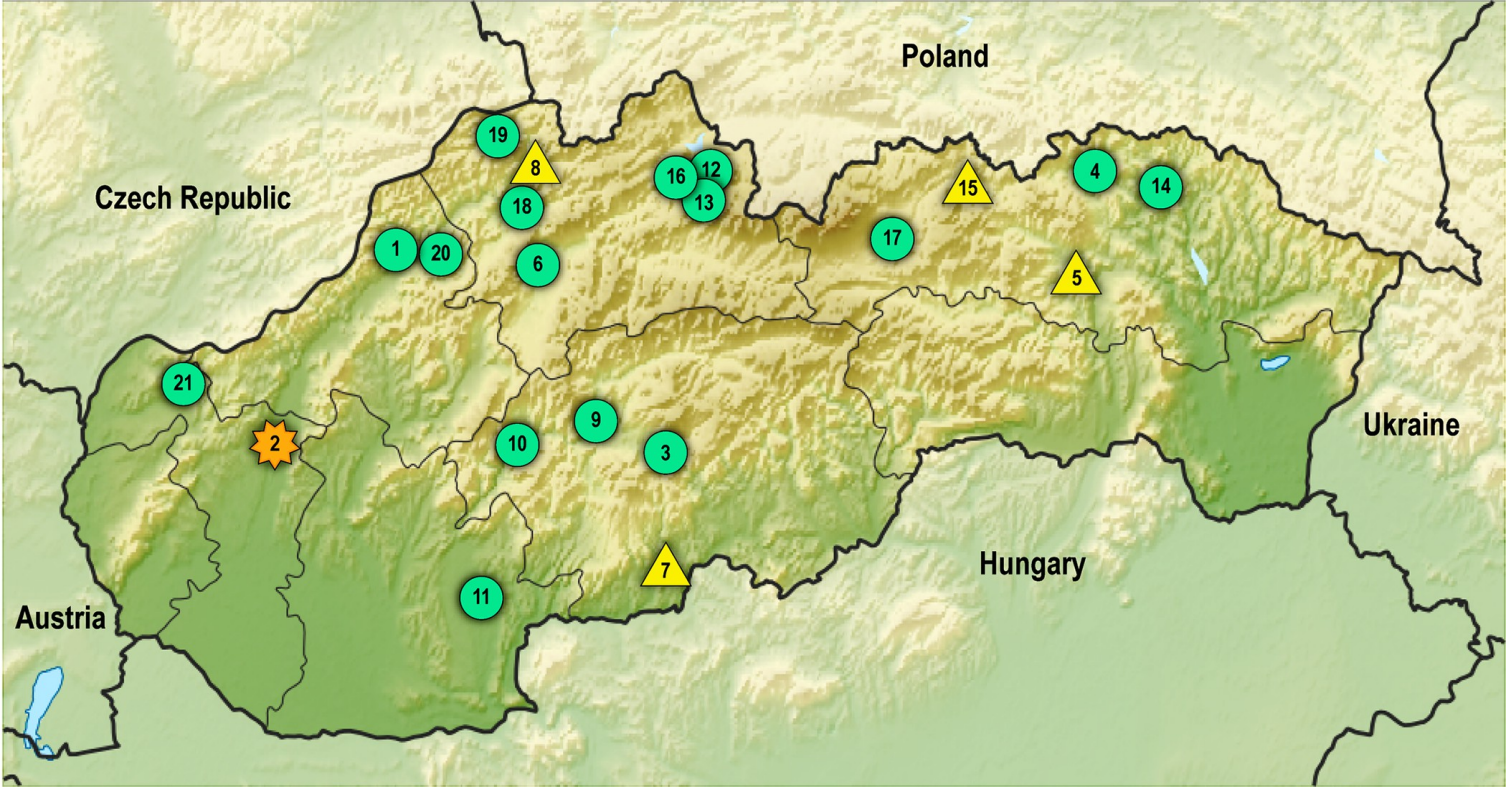
Geographic distribution of *Echinococcus multilocularis* haplotypes in Slovak patients. Green dots: E5-like profiles, yellow triangle: E4 profile, orange star: E1/E2 profile. Numbers correspond to patients´ IDs (e.g., No. 1 corresponds to SK1). The base map was used from public domain under a Creative Commons licence (PlaniGlobe, http://www.planiglobe.com.

Using concatenated data of *cob* (603 bp), *nad2* (882 bp), *cox1* (789 bp), which included a total of 2,274 sites of mitochondrial DNA, four haplotypes of *E*. *multilocularis* were detected in 21 samples from Slovak patients. Geographic distribution of haplotypes recorded using these markers is presented in Fig 4. Nucleotide bases detected in clinical samples in polymorphic sites of *cob*, *nad2*, and *cox1*, previously established for typing European E1 –E5 haplotypes [12], are listed in Table 3. In total, 16 isolates (76.2%) showed the E5-like profiles, 4 isolates (19%) the E4 profile and in 1 isolate (4.8%) the E1/E2 haplogroup was identified. Among the isolates assigned to E5, 14 isolates were entirely identical to E5 and two isolates (SK4, SK14) differed by a single nucleotide substitution (203 A/G) located in the *cox1* section, representing the variant designated here as E5^a^.

**Table 3:** Nucleotide bases in *cob*, *nad2* and *cox1* genes detected in Slovak isolates in polymorphic sites determined for European E1 –E5 haplotypes of *Echinococcus multilocularis* [12].

	*cob*	*cob*	603 bp	*nad2*	*nad2*	882 bp	*cox1*	*cox1*	*cox1*	*cox1*	789 bp	2,274 bp
**Isolate No.**	**244 G/T**	**369 A/G**	**Haplotype (*cob*)**	**72 A/G**	**486 A/G**	**Haplotype (*nad2*)**	**22 C/T**	**40 T/G**	**44 T/C**	**203 A/G**	**Haplotype (*cox1*)**	**Final Haplotype**
SK1	G	G	E5	G	G	E5	T	G	C	A	E2, E4, E5	E5
SK2	G	A	E1, E2, E4	A	A	E1, E2	–	–	–	–	–	E1/E2
SK3	G	G	E5	G	G	E5	T	G	C	A	E2, E4, E5	E5
SK4	G	G	E5	G	G	E5	T	G	C	G*	E2, E4, E5	E5^a^
SK5	G	A	E1, E2, E4	G	A	E3, E4	T	G	C	A	E2, E4, E5	E4
SK6	G	G	E5	G	G	E5	T	G	C	A	E2, E4, E5	E5
SK7	G	A	E1, E2, E4	G	A	E3, E4	T	G	C	A	E2, E4, E5	E4
SK8	G	A	E1, E2, E4	G	A	E3, E4	T	G	C	A	E2, E4, E5	E4
SK9	G	G	E5	G	G	E5	–	–	–	–	–	E5
SK10	G	G	E5	G	G	E5	T	G	C	A	E2, E4, E5	E5
SK11	G	G	E5	G	G	E5	T	G	C	A	E2, E4, E5	E5
SK12	G	G	E5	–	–	–	–	–	–	–	–	E5
SK13	G	G	E5	G	G	E5	T	G	C	A	E2, E4, E5	E5
SK14	G	G	E5	G	G	E5	T	G	C	G*	E2, E4, E5	E5^a^
SK15	G	A	E1, E2, E4	G	A	E3, E4	T	G	C	A	E2, E4, E5	E4
SK16	G	G	E5	G	G	E5	T	G	C	A	E2, E4, E5	E5
SK17	–	–	–	G	G	E5	–	–	–	–	–	E5
SK18	G	G	E5	G	G	E5	T	G	C	A	E2, E4, E5	E5
SK19	G	G	E5	G	G	E5	T	G	C	A	E2, E4, E5	E5
SK20	G	G	E5	G	G	E5	T	G	C	A	E2, E4, E5	E5
SK21	G	G	E5	G	G	E5	–	–	–	–	–	E5

The dash ("–") with a grey background indicates that no satisfactory amplification was achieved with this gene.

*Additional polymorphism (behind the referenced E1 –E5 polymorphism) in *cox1*.

Most patients infected with E5 (*n* = 11) were from the northern region of Slovakia characteristic by a predominant mountainous landscape, three isolates were recovered from the districts located in the Slovak Central Mountains in central Slovakia, one isolate from the southwest of Slovakia (northeastern part of the Danubian Upland), and one from the west part bordering the Czech Republic (Little Carpathians mountains). Both patients exhibiting the E5^a^ subtype with the single nucleotide polymorphism (SNP) were from the two adjacent districts in north-eastern Slovakia, Bardejov (SK4) and Svidník (SK14) (Fig 4). For five isolates (SK2, SK9, SK12, SK17, SK21), satisfactory results were not achieved after the PCR amplification of *cox1*; however, the obtained *cob* and *nad2* data were sufficient to categorize these samples as carrying the E5 haplotype (see Table 3). This was also the case of SK12 in *nad2* and SK17 in *cob*, which matched haplogroup E5 in the two remaining well-resolved gene fragments. It cannot be ruled out that the above five isolates could carry additional mutations beyond the sites differentiating major haplogroup variants; however, the haplogroup affiliation was consistent across gene fragments for all isolates.

The occurrence of haplotype E4 was dispersed throughout the country. Two of the patients (SK5, SK15) came from north-eastern Slovakia (districts of Sabinov and Stará Ľubovňa), one (SK7) from central-southern Slovakia (Veľký Krtíš district), and one (SK8) from the northwest of the country (Kysucké Nové Mesto district). For the SK2 isolate derived from western Slovakia (the only sample with metacestodes isolated from the brain lesion), satisfactory sequence profiles were achieved only for *cob* and *nad2* genes. Sequences obtained from these genes classified SK2 as belonging to the E1/E2 haplogroup.

**Fig 5:**
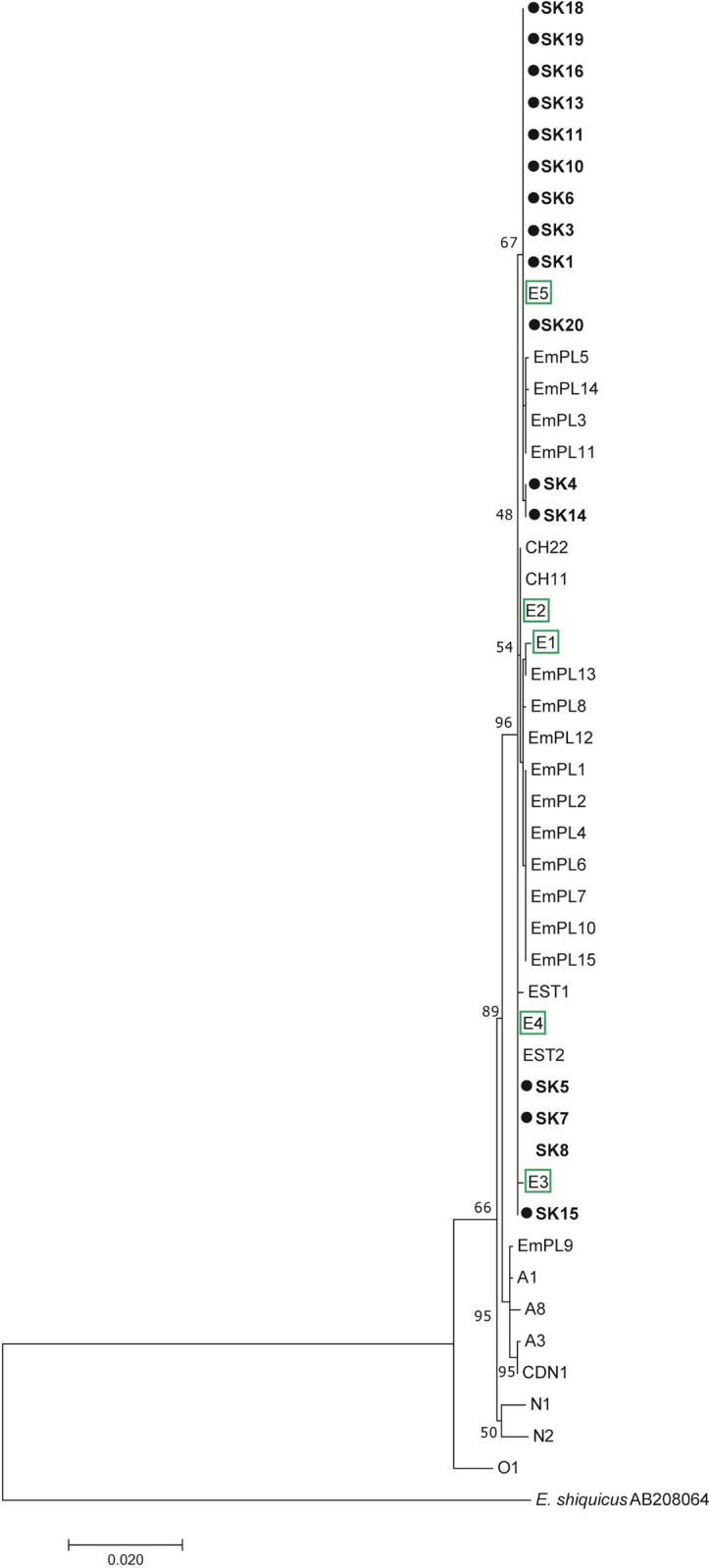
Maximum likelihood phylogenetic tree constructed using concatenated sequences of *cob1* (603 bp), *nad2* (882 bp) and *cox1* (789 bp), showing relationships between the examined Slovak isolates with completely recorded sequences for three genes and GenBank-related isolates of *Echinococcus multilocularis*. The scale bar refers to a phylogenetic distance of 0.0020 nucleotide substitutions per site. Geographical origins of the reference isolates: SK, Slovakia; PL, Poland; EST, Estonia; CH, Switzerland; CDN; Canada. E1-E5, reference isolates for Europe sensu Nakao et al. [12]; A1, A3, A8, reference isolates for Asia sensu Nakao et al. [12]; N1, N2, reference isolates for North America sensu Nakao et al. [12]. *Echinococcus shiquicus* (AB208064) from China was used as an outgroup.

**Fig 6:**
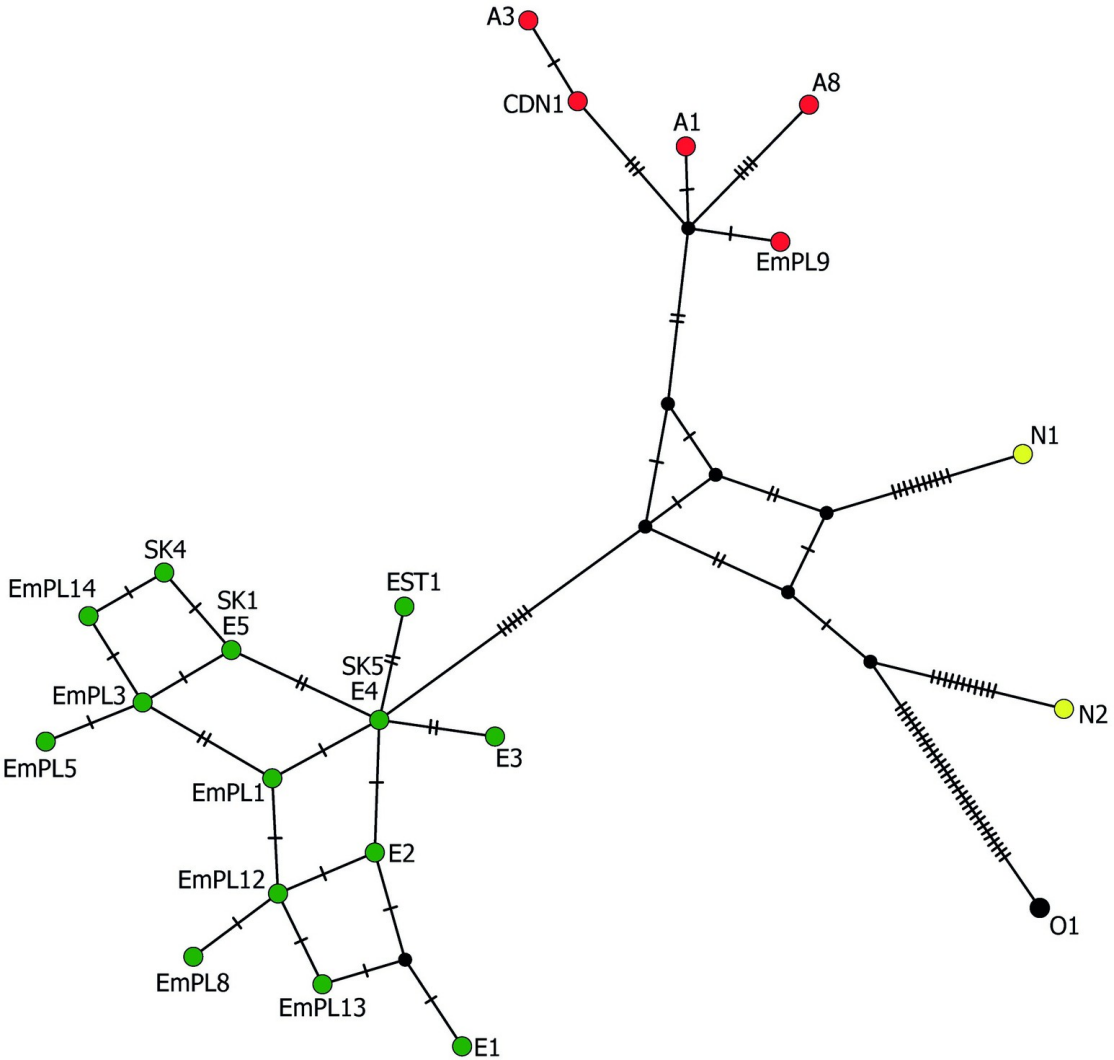
Median-joining haplotype network generated from concatenated sequences of *cob1* (603 bp), *nad2* (882 bp) and *cox1* (789 bp), illustrating relationships between the examined Slovak isolates with completely recorded sequences for three genes and related haplotypes of *Echinococcus multilocularis*. Labelled circles indicate representatives of distinct haplotypes, transversal bars at branches represent point mutations, and circle size correlates with haplotype frequency. Green circles–European haplotypes, red circles–Asian haplotypes, yellow circles–North American haplotypes. Sample codes: SK1, SK4, SK5 –examined isolates from Slovakia (this study); EMPL1, EMPL3, EMPL5, EMPL8, EMPL9, EMPL12, EMPL13, EMPL14 –isolates from Poland [33]; EST2 –isolate from Estonia [38]; CDN1 –isolate from Canada [39]; E1—E5, A1, A3, A8, N1, N2, O1 –reference isolates taken from Nakao et al. [12].

In the phylogenetic estimation conducted by the ML method inferred from concatenated sequences (Fig 5), the European *E*. *multilocularis* samples were separated from the North American and Asian samples, with the sister branch represented by the Mongolian sample O1. The branch consisting of European isolates was strongly supported (97%), comprising groups E1/E2 and E3/E4, and also included a moderately supported (67%) sub-branch composed of E5-like haplotypes. The average nucleotide divergence values among the E1/E2 and E3/E4 haplotype pairs (adopted from the Nakao´s scheme) were 0.06%. The E5 haplotype differed from the E1 –E4 variants from 0.08% (compared to E4) to 0.17% (compared to E1) of nucleotides. Within the samples assigned to three haplogroups (E1/E2, E3/E4, E5), none of the isolates differed by more than two mutational steps from one of the haplogroup members (as seen in the haplotype network in Fig 6).

According to available sources containing *cob*, *nad2* and/or *cox1* sequences (listed in S3 Table), we have derived that the haplogroup E1/E2 was documented in France (*n* = 4), Switzerland (*n* = 2), Austria (*n* = 1), Germany (*n* = 1), Slovakia (*n* = 1), and Poland (*n* = 7). The haplogroup E3/E4 was resolved in France (*n* = 6), Germany (*n* = 5), Belgium (*n* = 2), Poland (*n* = 51), Slovakia (*n* = 4), Estonia (*n* = 9), and Latvia (*n* = 1). The haplogroup formed around E5 was detected only in Slovakia (*n* = 22) and Poland (*n* = 9).

From the sequences in the above three genes yielded from 74 red foxes in Poland (neighboring to Slovakia from north), provided by Karamon et al. [33], we estimated that the haplogroup E3/E4 can be attributed to 68.9% of Polish foxes (51/74), the haplotype E5 to 12.1% of foxes (9/74), the haplogroup E1/E2 to 9.5% of foxes (7/74), and in 9.5% of foxes (7/74) the Asian haplotype was found. A difference between dominant haplogroups in Slovakia (E5) and Poland (E3/E4) was thus recorded, despite the fact that a large part of the samples was investigated from the areas of southern Poland and northern Slovakia connected by the continuous flysch zone of the Outer Western and Inner Western Carpathians. When the compared zones were restricted to southern Poland and northern Slovakia, the same predominant haplogroups were determined for Poland (E3/E4, a frequency of 57.4%, 19/33) and Slovakia (E5, a frequency of 78.5%, 11/14), although in southern Poland the E3/E4 cluster was not as prevailing as it was throughout the whole country.

### Diversity and neutrality indices for *E*. *multilocularis* populations

A population genetic structure was evaluated in isolates collected from 16 Slovak patients with the yielded sequences in all three gene sections (*cob*, *nad2*, *cox1*), along with the reference 74 samples from Poland [33] and 10 samples from France [12]. Analyses of the *cob*, *nad2* and *cox1* sequences indicated a high level of the overall haplotype diversity (total Hd: 0.716), while the overall nucleotide diversity was relatively low (total Nd: 0.00117) (Table 4). The population derived from Poland appeared to be more diverse with regard to the nucleotide diversity and the average number of nucleotide differences, showing approximately twice higher values (Nd: 0.00104, K: 2.356) compared to populations from Slovakia (Nd: 0.00045, K: 1.033) and France (Nd: 0.00041, K: 0.933). This was largely due to the eight haplotypes (assigned to four haplogroups), including a more diverged Asian haplotype, found in Poland, compared to three haplotypes considered for the diversity estimations in Slovakia and France. A higher number of evaluated samples in Poland retrieved from public repositories may also have contributed to this difference.

**Table 4:** Diversity and neutrality indices for *Echinococcus multilocularis* populations from Slovakia, Poland and France derived from *cob1*, *nad2* and *cox1* data.

		Diversity indices					Neutrality index		Simpson´s index (1-D)
	n	Hn	Hd ± SD	Polymorphic sites (ts/tv)	Nd ± SD	K	Tajima´s D	P value	
**Poland**	74	8	0.513 ± 0.00460	16 (11/5)	0.00104 ± 0.000636	2.356	-0.82308	P > 0.10 (ns)	0.51
**Slovakia**	16	3	0.567 ± 0.01187	3 (3/0)	0.00045 ± 0.000358	1.033	0.41440	P > 0.10 (ns)	0.57
**France**	10	3	0.644 ± 0.01025	3 (2/1)	0.00041 ± 0.000347	0.933	-0.43410	P > 0.10 (ns)	0.64
**Total**	100	13	0.716 ± 0.04516	19 (12/7)	0.00117 ± 0.000175	1.441	-0.79397	P > 0.10 (ns)	0.72

n, number of isolates; Hn, number of haplotypes; Hd, haplotype diversity; ts/tv, transitions/transversions; Nd, nucleotide diversity; K, average number of nucleotide differences

Unlike that, haplotype diversity and Simpson´s Diversity Index (1-D) values were higher in the French population compared to Slovakia and Poland. In French samples, five E4 haplotypes, four E2 haplotypes and one E3 haplotype (the singleton sequence within the entire dataset) were detected by Nakao and colleagues in 2009 [12].

When *E*. *multilocularis* populations were treated as one group, the overall Tajima’s D showed a negative value (-0.79397), statistically not significant (P > 0.10), suggesting no overall substantial divergence from neutrality. When accounting for separated populations by country, the Tajima’s D for Slovakia gave a positive value (but not significant), due to a more balanced distribution of three haplotypes considered for the population dataset. This value was, however, biased by the absence of the singleton E1/E2 sequence for Slovakia, which was omitted for this evaluation due to the absence of the *cox1* data, and would have influenced Tajima´s D towards negative values (if considered). Negative values of Tajima´s D obtained in remaining populations indicated that there is an excess of rare mutations, which can imply the recent population expansion; however, the excesses were not statistically significant.

### Genetic differentiation between *E*. *multilocularis* populations (pairwise Fst values)

When genetic subdivision among *E*. *multilocularis* populations from Slovakia, Poland and France was measured by pairwise Fst indices, ranges from 0.3363 to 0.5546 were obtained (Table 5). Very high Fst indices (Fst > 0.25) as estimated here indicate a large degree of genetic differentiation and separation between pairs of populations. Paradoxically, populations from neighbouring Slovakia and Poland (where some gene flow between tapeworms close to bordering areas might occur) showed greater genetic divergence (Fst = 0.4589) than populations from the more distant countries France and Poland (Fst = 0.3363). This is mainly due to that the same predominant haplogroup E3/E4 was recorded in France and Poland (in frequencies of 60% and 68.9%), and the more distinct Asian haplotype in Poland (and absent in Slovakia). The highest genetic subdivision (Fst = 0.5546) was measured between populations from France and Slovakia.

**Table 5:** Pairwise fixation indices (Fst) for *Echinococcus multilocularis* populations from Poland, Slovakia and France calculating from *cob1*, *nad2* and *cox1* data.

	1	2	3
1 POL	-		
2 SK	0.4589*	-	
3 FRA	0.3363*	0.5546*	-

*Statistically significant values (P < 0.05)

### DNA sequencing of nad1

**Fig 7:**
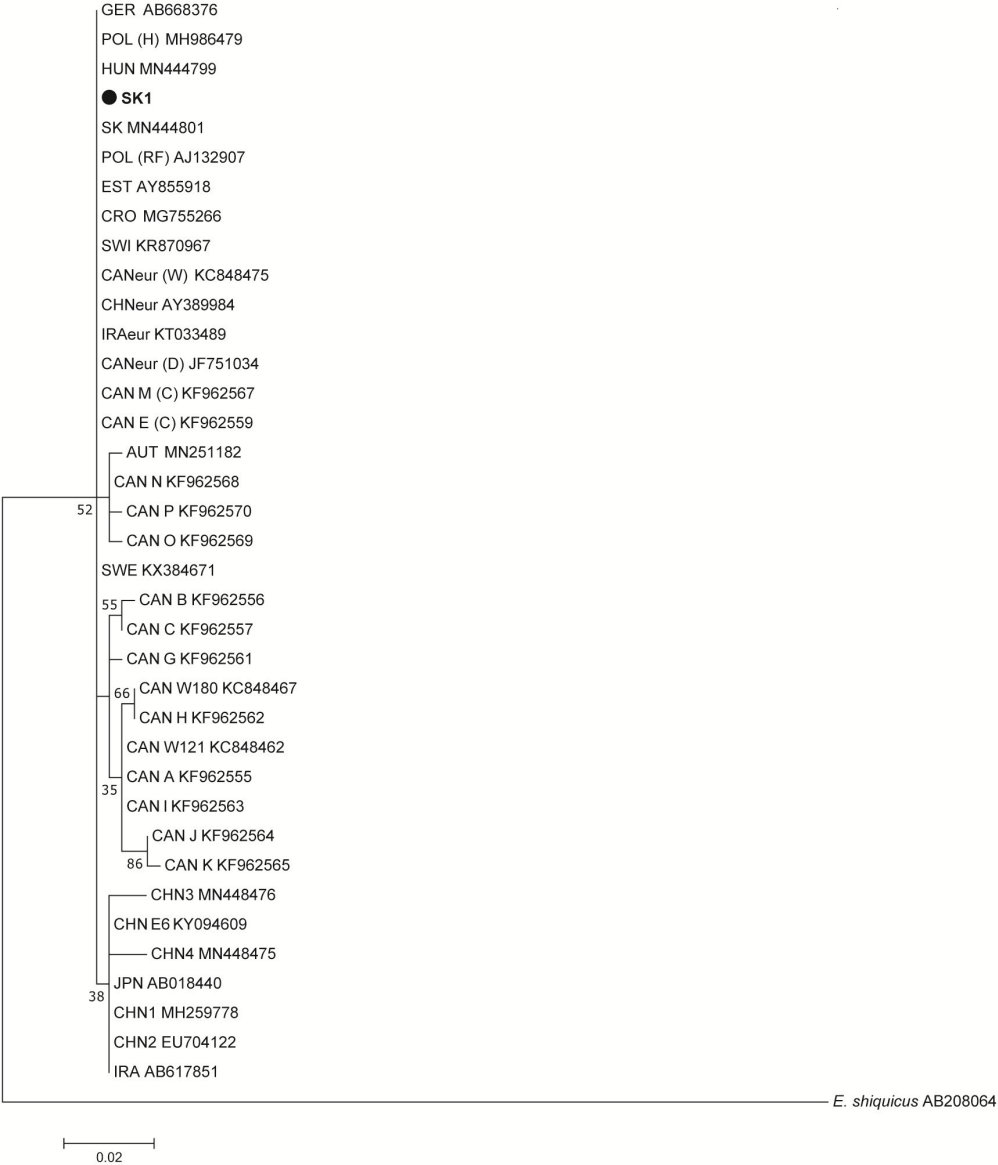
Maximum likelihood phylogenetic tree constructed using sequences of *nad1* (395 bp), showing relationships between the examined Slovak isolates and GenBank-related isolates of *Echinococcus multilocularis*. The scale bar refers to a phylogenetic distance of 0.0010 nucleotide substitutions per site. Geographical origins of the reference isolates: SK, Slovakia; HUN, Hungary; GER, Germany; POL, Poland; EST, Estonia; CRO, Croatia; SWI, Switzerland; CAN, Canada; CHN, China; IRA, Iran; AUT, Austria; JPN, Japan; SWE, Sweden. Hosts abbreviations in parentheses: (H), human; (RF), red fox; (W), wolf; (D), domestic dog; (C), coyote. *Echinococcus shiquicus* (AB208064) from China was used as an outgroup.

Twenty-one samples of *E*. *multilocularis* obtained from the same Slovak patients (listed in Table 1), subjected to the analysis of the partial *nad1* gene (395 bp), yielded positive PCR and sequence results. A typical European haplotype with a monomorphic pattern was found in all examined samples. In the ML phylogram the examined and reference samples clustered mainly according to continental origin (European, Asian and North American subgroups), without more robust statistic support given the low amount of variation at this molecular marker (Fig 7).

Identical sequences to our samples were found in three isolates from red foxes that were studied earlier in Slovakia [40], in 11 isolates (six from red foxes, five from humans) in Poland [40–42], in six isolates from red foxes in Estonia [27], in four isolates from red foxes in Sweden [43], in two isolates from red foxes in Hungary [40], in one isolate from patient in Croatia [24], in one isolate from European brown hare in Switzerland [44], and in one isolate from the Barbary macaque originating from a wildlife park in Germany (Genbank entry AB668376).

Outside of Europe, identical sequences assigned to the major European haplotype were also detected in eight isolates from golden jackals in northeastern Iran (North-Khorasan Province), in contrast to two fox isolates from the same area that carried the Asian haplotype [45]. The European-like profile was recorded also in China, Xinjiang (GenBank entry AY389984). Elsewhere, identical sequences to this pattern were classified as a European-type haplotype in surveys conducted in western and central Canada on *E*. *multilocularis* from coyotes, wolves, foxes and the domestic dog [11,46,47].

**Fig 8:**
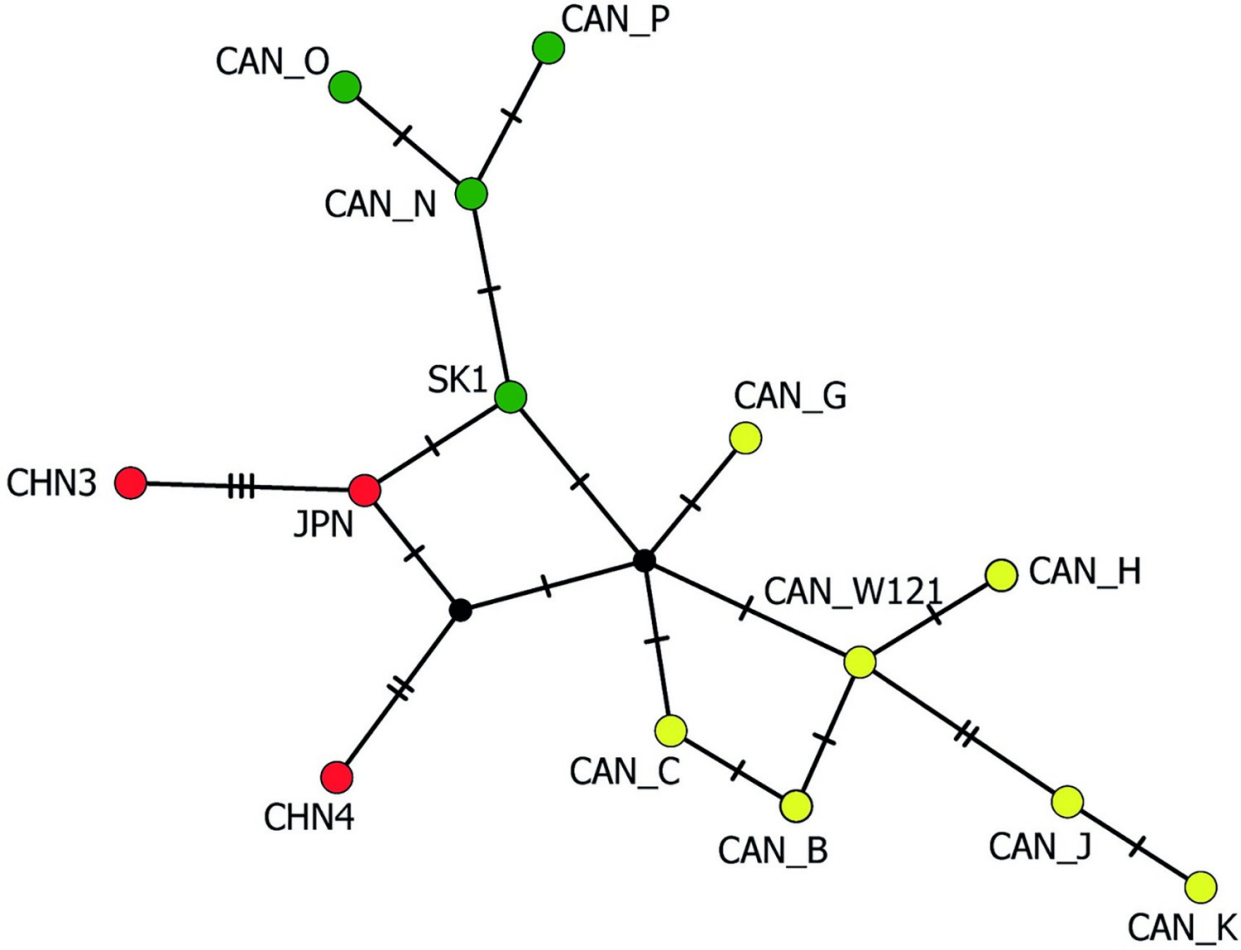
Median-joining haplotype network generated from sequences of *nad1* (395 bp), illustrating relationships between the examined Slovak isolates and related haplotypes of *Echinococcus multilocularis*. Labelled circles indicate representatives of distinct haplotypes, transversal bars at branches represent point mutations, and circle size correlates with haplotype frequency. Green circles–European haplotypes, red circles–Asian haplotypes, yellow circles–North American haplotypes. Sample codes: SK1 –examined isolate from Slovakia; CAN-N, CAN-O, CAN-P–isolates from Canada with European profiles [11]; CHN3, CHN4 –isolates from China (GenBank Nos. MN448475 and MN448476); JPN–isolate from Japan [48]; CAN-W121, CAN-B, CAN-C, CAN-G, CAN-H, GAN-J, CAN-K–isolates from Canada with North American profiles [11,47].

The less frequent haplogroup located in the European cluster (a bootstrap value of 52%) consisted of an older Austrian clinical isolate recovered in 1981 [40] and Canadian haplotypes, designated as CAN-N, CAN-O, CAN-P by Gesy et al. [11]. These samples are genetically closer to the European group than to the Asian group, as seen in the haplotype network constructed for *nad1* (Fig 8). They exhibited the average genetic identity of 99.5% to the European cluster and 99.2% identity to the Asian cluster.

### Availability of DNA sequence data

The nucleotide sequences of 20 isolates detected in *cob* (603 bp) were deposited in GenBank under accession numbers OP277487-OP277506. The sequences of 19 isolates detected in *nad2* (882 bp) were deposited under accession numbers OP277507 –OP277525. The sequences of 16 isolates detected in *cox1* (789 bp) were deposited under accession numbers OP225398, OP225402, OP225448, OP225555, OP225644, OP225830 and OP225945-54. The sequences of 21 isolates detected in *nad1* (395 bp) were deposited under accession numbers MW326786-7, MW343787-9, MW357715, MW366778-79, MW384819-20 and OP356581-OP356591.

## Discussion

In the current study, data on 137 AE patients reported between January 2000 and October 2023 in Slovakia are presented. The very first case of human AE in the country had been described by Krčméry et al. in 1989 [49] in a patient from western Slovakia hospitalized in 1988. The clinical picture showed an infiltrative process in liver and high level of antibodies against *Echinococcus* spp. Computer tomography revealed numerous smaller vesicles and calcifications in the diffusely damaged part of the liver, suggestive of AE. After a decade, the other two cases were diagnosed in 2000 in the Žilina region (north-western part of the country) in 69- and 71-year-old women [50,51]. In the first case, the dyspeptic symptoms accompanied by pain in the right epigastrium appeared already in 1994, suggesting development of infection before 2000. In 2004, two other cases appeared and since then, new cases have been reported every year. By 2011, one to four cases of the disease were confirmed every year (annual incidence 0.031/100,000 inhabitants). Since 2012, the number of diagnosed patients has increased to a level from 7 to 15 per year, corresponding to the annual incidence of 0.187/100,000 inhabitants. Similarly, the rise of patients’ numbers was recorded in historically endemic areas in Europe, where the AE incidence has doubled since 2000 [52], and continual spread of infection throughout the Europe confirmed the occurrence of new AE cases in most countries of central and eastern Europe, the Baltic states, as well as in previously non-endemic areas as Belgium and Netherlands [53,54].

Several authors reported differences in AE incidence among countries or districts, as well as changes over time. In the region of Vorarlberg, Austria, the annual incidence of infection per 100,000 inhabitants rose from 0.08 in the period of 1991–2000 to 0.32 between 2001 and 2010, but in the same time periods it decreased from 0.17 to 0.07 in the Tyrol region [54]. In Canton of Fribourg in Switzerland, the average annual incidence increased from 0.1 in 1993–2000 to 0.26 in 2001–2005 [55], while in France, a relatively stable incidence of AE ranging from 0.023 to 0.026 was reported between 1982 and 2009 [56,57]. In Poland, striking differences between Warmia-Masuria Province located in the northeast of the country (0.2/100,000 inhabitants/year) and the rest of Poland (0.014/100,000 inhabitants/year) were reported by Nahorski et al. [58]. In this study, the differences among districts with higher or lower prevalence of the tapeworm were quite apparent. Most of the patients, 75 out of 95 with the available information on residence (78.9%; incidence of 0.09/100,000 inhabitants/year), came from the northern regions of Slovakia (Trenčín, Žilina and Prešov regions), where the prevalence of *E*. *multilocularis* in red foxes ranged between 39.1% and 49.6%, and in some districts even overreached 60%. These regions are characterized by low mean annual air temperature (4–8° C), high mean annual precipitations (700–1,300 mm), high soil humidity and low mean annual soil surface temperature (3–7°C) which support long-term survival of tapeworm eggs in the environment. On the other hand, for areas situated towards the south of Slovakia (Bratislava, Trnava, Nitra, and Košice regions), warm and dry climate, with high mean annual air temperature (8–12°C), low mean annual precipitations (450–680 mm), and higher mean annual temperature of soil surface (10–12°C), is characteristic [59]. In these southern parts of the country, the prevalence of *E*. *multilocularis* in red foxes varied between 11.5% and 24.8%, and only 20 patients (21.1%; incidence of 0.03/100,000 inhabitants/year) settled herein. The spread of AE among inhabitants is probably also related to the high population density of red foxes reported throughout the Slovakia [60], which in turns leads to higher contamination of the environment with parasite eggs.

According to the available data, the greatest increase in the Slovak fox population over the last decades occurred between 1993 and 1996. In 1993 the stock fox population was estimated to be 6,154, but in 1996 it was 13,331, i.e. more than double the amount [61–63]. In the 2021/2022 hunting season, the spring stock of game in the hunting grounds was reported to be 36,927 foxes [60], which corresponds to a significant, about six-fold increase compared to 1993. The dispersion of foxes was documented in a substantial part, 93.5%, of the Slovak territory [64]. The increased population density of foxes is largely connected with the launch of full-range anti-rabies vaccination of foxes in 1994 with a subsequent reduction in mortality and a decrease of hunting pressure due to e.g. the decrease in the price of fox fur since the 1990s [65]. The parasite practically did not occur in the territory of Slovakia in the 1960s, 1970s and 1980´s based on regular detailed surveys of the parasitic fauna of wild animals and rodents, which failed to find any mature or larval stages of *E*. *multilocularis* [e.g., 66–69]. The single above human AE case registered in the late 1980´s indicate that some endemic foci could be established in Slovakia in this decade due to the continuous migration of young foxes from historical endemic areas with increased population densities of foxes established primarily due to their anti-rabies vaccinations, the first of which was commenced in Switzerland in 1978 [70].

Our data on the location of metacestode lesions in patients infected with AE are consistent with the majority of published records referring that hepatic involvement is dominant, and about 90% of the lesions are found in the liver [71]. In this study, in 86.4% of patients the liver was the only affected organ and in 12 cases spread to other organs was reported. In the 8-year-old boy, the primary affected organ was left femur, but after a period of time, the liver involvement occurred.

In the present study, a total of four haplotypes of *E*. *multilocularis* collected from 21 selected Slovak patients were recorded. Most of the analyzed samples were derived from the typically mountainous area of the North-Carpathian arc, which stretches throughout the northern part of Slovakia. The highest number of samples subjected to DNA analyses were from patients coming from the regions of Žilina (7 cases) and Prešov (5 cases), which are the areas with the highest prevalence of parasite in foxes (49.6%, 39.1%) and mean worm burdens (2,070 and 2,515 adults, respectively), as summarized by Miterpáková and Dubinský in 2011 [8]. Seventeen patients were originating from the Western Carpathians, 2 patients from the Eastern Carpathians (two easternmost samples from the Low Beskid mountains), and 2 patients from lowland areas. Among the geomorphological subprovinces, the Outer Western Carpathians, which constitute the northern rim of the country, were the most sampled area (8 samples; 6xE5, 2xE4), followed by the Inner Western Carpathians (7 samples; 5xE5, 2xE4), 2 samples were from the edge area of the Outer and Inner Western Carpathians (2xE5), 2 samples from the Low Beskids in the Outer Eastern Carpathians (2xE5 subtype) and 2 samples (E5, E1/E2) were from the Danubian Lowland (Fig 4).

The E5-type haplotypes were the most abundant (16/21, 76.2%) and distributed throughout the country. The easternmost sampled isolates (SK4, SK14) from the adjacent districts (Bardejov, Svidník) carried the E5^a^ subtype exhibiting the substitution 203 A/G, the variant apparently dispersed within the area. Interestingly, only E5-like variants with 1–2 nucleotide differences (EmPL3, EmPL5, EmPL11, EmPL14) compared to the typical E5 pattern (highly prevailing in Slovakia) were established in Poland [33], despite the feasible transborder movement of foxes between Poland and Slovakia (except for the south of Nowy Targ district, where the High Tatras form a natural barrier). The area-specific clusters with E5 variants may have been differentiated by genetic drift due to partial isolation for generations in a mountainous landscape of southern Poland.

Two of the four isolates corresponding to E4 haplotypes (SK5, SK15) were recovered from patients coming from adjacent districts (Sabinov, Stará Ľubovňa) in eastern Slovakia located in the Podhale-Magura Area and the Eastern Beskids geomorphological regions (eastern border of the Outer Western Carpathians). Cestodes belonging to the E3/E4 haplogroup were shown to be common in the adjacent territory of southern Poland, where they were recorded in 57.4% of fox isolates by Karamon et al. in 2017 [33]. The two remaining E4-typed samples were originating from the isolated foci in south-central Slovakia and north-central Slovakia.

According to the data so far gathered in *cob*, *nad2* and *cox1* genes for Europe, the haplogroup E1/E2 of *E*. *multilocularis* was before recorded in Switzerland and Austria (as an exclusive genetic variant), and in France, Germany, Poland and Slovakia (as a minor variant) [12,33,39]. The haplogroup E3/E4 was recorded in France, Germany and Poland (as a major variant), Belgium, Estonia and Latvia (as an exclusive variant), and Slovakia (as a minor variant) [12,38,40,72,73], i.e. obviously in the area covering more northern territory (including the historical endemic focus of the northern Alpine region) than the area affiliated with the E1/E2 distribution. Haplotypes corresponding to the haplogroup E5 were documented only in Slovakia (as a major variant) and Poland (as a minor variant) [12,33]. The E5 dispersal thus seems to be restricted to central-eastern Europe, being historically indigenous to this region, and reflecting a deeper historical pattern related to the postglacial expansion. During the postglacial colonization in the mid Holocene (8.2–4.2 kya), red foxes had expanded over most of their present territory and formed continuous populations across central and eastern Europe [74,75], thus playing an important role in the current distribution and genetic diversity of the parasite [12].

For Slovakia, Nakao et al. [12] detected a uniform sequence pattern in three genes, in which all 10 geographical isolates from foxes sampled in the period 2004–2006 showed a typical E5 structure. Unlike this, in 21 human samples from the present study retrieved in the period 2013–2021, in addition to the two haplotypes with E5 type profiles (76.2%), four (19%) isolates exhibited the E4 profile and one isolate (4.8%) showed the E1/E2 profile, suggesting that admixture may have occurred from the historical endemic focus, where the two latter genetic types predominate, between the two samplings. Based on the currently known distribution of the major genetic types, it is probable that E1/E2 was introduced to Slovakia from a more southern zone of the historical endemic region (likely candidates are Switzerland, Austria) and E4 was transmitted from the region further north (the likely route was from the Swabian Jura in southern Germany, then through Bavaria and the Czech Republic).

Relatively high values of haplotype diversity (Hd: 0.567) were calculated in the present study for the clinical Slovak samples as well as for the entire dataset consisted of populations from Poland, Slovakia and France (Hd: 0.716). This overall value is similar to that assessed globally (Hd: 0.704) by Spotin et al. [76] for human-derived *E*. *multilocularis* using the *cox1* gene. Greater values of haplotype diversity and Simpson´s diversity were herein estimated for population sampled in France (derived mainly from the historically documented endemic focus) compared to Slovakia and Poland, where a higher transmission intensity and a more balanced genotype distribution of the longer-established population had supposedly an impact in increasing these values.

Interestingly, Slovak tapeworms were found to be more genetically differentiated to Polish worms than to French worms as was estimated in the present study by pairwise fixation indices, despite the partial sharing of mountainous Carpathian region with specific climatic conditions. Unlike Slovakia with a striking dominance of the E5 haplotype, the E4-type haplotypes prevail in Poland, including the southern part of the country (especially in the Upper Silesia bordering the Czech Republic), and are very dominant in the western and central parts of Poland. Furthermore, approximately 8–9.5% of Polish *E*. *multilocularis* showed profiles clustering with samples of Asian origin in DNA sequencing and microsatellite studies [33,77]. The influence of foxes (and raccoon dogs) from the countries situated in the north-east of Poland has been assumed in several studies, which indicated that northern Poland may serve as an important movement corridor for the tapeworm in both directions, from the historical northern Alpine region and from the Baltic/eastern countries [38,78,79], and is consistent with the previous hypothesis of a connection between the central European endemic focus and a large endemic focus, which extends from Siberia and the Russian Far East [80].

In the previous studies addressing the genetic diversity of *E*. *multilocularis* in Slovakia by DNA sequencing, 10 isolates from wolves and four isolates from dogs from five locations in eastern and central Slovakia were examined by employing *nad1* and 12S RNA genes [16,17]. All samples were assigned to the European haplotype of *E*. *multilocularis*, although two singletons with one SNP were detected in both genes in the two wolf isolates from the Poloniny National Park situated on the borders with Poland and Ukraine. Three fox isolates from Slovakia (one each from eastern, central and western Slovakia) were also examined in four mitochondrial (*cox1*, *nad1*, *atp6*, 12S rRNA) and one nuclear gene (*actII*) as a part of a wider European study, and yielded characteristic European profiles [38]. Further, Šnábel et al. [14] confirmed congruence with characteristic European patterns in 12 fox samples from 11 districts located across the country using *cox1* and allozyme markers. The *nad1* fragment screened in this study in 21 patients was identical to previously detected patterns in Slovakia derived from four wolves, three foxes, three dogs [14,16,17], with the exception of one wolf from the Poloniny biosphere reserve infested with a tapeworm carrying a nucleotide substitution G/A [17]. The nearest sites of the examined patients to this area were located around 100 km in the districts of Sabinov (sample SK5) and Svidník (sample SK14).

*E*. *multilocularis* seems to be on the rise in central-eastern Europe. In Slovakia the average annual human incidence rate of AE has increased about six-fold between the periods of 2000–2011 and 2012–2023. A significant positive spatial correlation was found between incidence of human AE and prevalence of parasite in foxes since 2000. The relatively high haplotype diversity (four DNA profiles found in 21 patients) observed at the discriminatory loci may contribute to a better understanding of the distribution of regional profiles as well as differentiating range expansions of genetic types. It is needed to further genetically characterize *E*. *multilocularis* populations in other hosts (red foxes, raccoon dogs, jackals, wolves) throughout the territory of Slovakia to better recognize patterns of the parasite spread in relation to neighboring countries.

## Supporting information

S1 Table Data on human patients with alveolar echinococcosis diagnosed in Slovakia, January 2000 –October 2023. (XLSX)

S2 Table Data on incidence of human alveolar echinococcosis and prevalence of *Echinococcus multilocularis* in red foxes in different districts of Slovakia. (XLSX)

S3 Table Records of *Echinococcus multilocularis* corresponding to European haplogroups E1/E2, E3/E4 and E5. (XLSX)
